# Risk factors and clinical impact of bone pain in patients with multiple myeloma – A prospective interdisciplinary study

**DOI:** 10.1016/j.jbo.2026.100788

**Published:** 2026-07-21

**Authors:** Carlotta Pietsch, Monika Engelhardt, Julian P. Maier, Gabriele Ihorst, Hagen Schmal, Evangelos Terpos, Ioannis Ntanasis-Stathopoulos, Ralph Wäsch, Georg W. Herget

**Affiliations:** a Department of Orthopedics and Trauma Surgery, Medical Center – University of Freiburg, Faculty of Medicine, Freiburg, Germany; b Comprehensive Cancer Center Freiburg (CCCF), Medical Center - University of Freiburg, Faculty of Medicine, Freiburg, Germany; c Department of Medicine, Hematology and Oncology, Medical Center – University of Freiburg, Faculty of Medicine, Freiburg, Germany; d Clinical Trials Unit, Medical Clinic, Medical Center – University of Freiburg, Faculty of Medicine, Freiburg, Germany; e Department of Clinical Therapeutics, School of Medicine, National and Kapodistrian University of Athens, Athens, Greece

**Keywords:** Multiple myeloma, Bone pain, Osteolysis, Myeloma bone disease, Orthopedic condition, PROMs

## Abstract

**Background:**

Bone pain affects up to 80% of patients with multiple myeloma (MM) patients and significantly impairs quality of life (QoL), yet its specific predictors remain poorly characterized. Identifying risk factors is essential for early intervention and personalized treatment.

**Methods:**

This prospective, single-center study included 352 patients. Bone pain was recorded using a standardized questionnaire. Multivariate logistic regression with stepwise selection was used to identify independent predictors of bone pain. QoL and health-related status were assessed on a 7-point scale and compared using the Wilcoxon two-sample test.

**Results:**

Bone pain was reported by 184 patients (52%). Independent predictors of bone pain were osteolytic lesions with fracture risk (OR 3.06, 95% CI 1.46–6.40, *p* = 0.003) and pre-existing orthopedic disease (OR 2.78, 95% CI 1.77–4.36; *p* < 0.0001). CRAB (hypercalcemia, renal failure, anemia, bone lesions)-B at initial diagnosis (OR 1.74, 95% CI 0.92–3.29, *p* = 0.09) and progressive disease (OR 1.68, 95% CI 0.93–3.05, p = 0.09) showed an elevated risk for bone pain. No associations were observed for age, sex, CRAB-C/-R/-A, International Staging System (ISS) stage, and disease duration after adjustment. Patients with bone pain had significantly lower median QoL and health-related status compared to patients without pain (*p* < 0.001).

**Conclusions:**

Osteolytic lesions with fracture risk and pre-existing orthopedic conditions independently predict bone pain in patients with MM. These findings highlight the different etiology and the overlap between orthopedic and oncological conditions. Interdisciplinary management is essential to distinguish MM-caused pain from degenerative pain and optimize individualized care.

## Highlights

• Osteolytic lesions with fracture risk triple the odds of bone pain.
• Pre-existing orthopedic conditions independently predict bone pain.
• Bone pain affects about half of all patients and significantly reduces quality of life.
• One-third of bone pain cases did not correlate with myeloma skeletal lesions, suggesting substantial non-malignant contributions.
• An interdisciplinary evaluation is needed to differentiate pain etiology and optimize treatment strategies.

## 1 Introduction

Multiple myeloma (MM) is the second most common malignant hematologic neoplasm [1], [2]. It is characterized by the clonal proliferation of malignant plasma cells within the bone marrow. These myeloma cells disrupt the physiological balance between osteoclasts and osteoblasts during bone metabolism, leading to increased bone resorption and impaired new bone formation [1], [3]. As a result, patients exhibit bone destruction like secondary osteoporosis and osteolytic lesions, which in severe cases progress to pathological fractures and other skeletal-related events (SREs) [4], [5]. As a result, bone pain is a hallmark symptom, affecting up to 80% at diagnosis [6], [7], [8].

It is important to understand that bone disease is not just a physiological problem, but profoundly impairs patients' daily function, although the burden of bone disease may not impact overall survival outcomes [9]. Recent studies on Patient-Reported Outcome Measures (PROMs) in MM have shown that bone pain is one of the main factors contributing to reduced Quality of life (QoL) [10], [11], [12].

However, while osteolytic lesions are common [13], not all patients experience bone pain to the same extent, suggesting that additional co-existing factors may contribute to the development or severity of bone pain symptoms [7]. Despite the high prevalence and clinical significance of bone pain in MM, comprehensive prospective studies examining independent predictors remain scarce.

The primary objective of this prospective study was to identify independent clinical and demographic predictors of bone pain in a large cohort of MM patients using multivariable analysis. Secondary objectives included assessing the impact of bone pain on patient-reported QoL and health-related status.

## 2 Materials and methods

### 2.1 Study design and patient selection

A total of 352 follow-up patients with MM were included in the prospective single-center study. The main inclusion criterion was a confirmed diagnosis of MM. Exclusion criteria for participation included: newly diagnosed MM patients, monoclonal gammopathy of unknown significance (MGUS), smouldering multiple myeloma (SMM), exclusive antiresorptive therapy (denosumab or bisphosphonates) any debilitating medical or mental condition that affected a patient's consciousness, and the inability to provide written informed consent for our thorough assessment. All eligible patients who presented during the study period from February 2022 to June 2024 were consecutively included. If patients attended multiple visits during the recruitment period, each visit was considered a separate study observation.

Our primary objective was to evaluate clinical and disease-related risk factors for the occurrence of bone pain, defined as a binary variable (yes/no) at the time of assessment. The secondary objective was to evaluate the influence of bone pain on PROMs.

For the interview-based analysis, a standardized questionnaire was administered to patients during their routine follow-up at the outpatient clinic of the Comprehensive Cancer Center Freiburg (CCCF), University Medical Center Freiburg. The questionnaires were distributed at a random time point to eligible patients, taking the predefined exclusion criteria into account, regardless of (previous) treatments (drugs, type of current therapy, radiotherapy, etc.). The questionnaire included demographics (age at study inclusion, age at ID, sex), disease characteristics: CRAB (hypercalcemia, renal failure, anemia, bone lesions), International Staging System (ISS) stage, duration of disease, osteolysis with risk of structural instability at study inclusion, assessed by computed tomography (not older than 3 months), current MM-therapy, pre-existing orthopedic conditions, occurrence of skeletal pain, and Patient-Reported Outcome Measures (PROMs), including QoL and health-related status. The latter two were assessed using a scale ranging from 1 (very poor), 2 (poor), 3 (fairly poor), 4 (neutral), 5 (fairly good), 6 (good), to 7 (excellent). Patient-reported pre-existing orthopedic conditions were systematically reviewed and medically validated by the study physicians. Self-reported information was assessed in conjunction with the available medical records, imaging findings (including degenerative changes and osteolytic lesions), and, where available, the clinical examination performed by the study team. Osteolytic lesions considered at risk of instability were identified retrospectively from routine clinical radiological reports and interdisciplinary clinical documentation based on CT imaging obtained within three months of study inclusion. No dedicated study-specific re-evaluation of CT scans, formal instability scoring system, or central blinded radiological review was performed.

The study was conducted following the guidelines of the Declaration of Helsinki and Good Clinical Practice. All patients provided written informed consent for institutional-initiated research studies and analyses of clinical outcome studies, in accordance with our institutional review board guidelines (Ethical committee No. 30/18_250091).

### 2.2 Data sources for demographic and clinical information

Patient and disease characteristics were collected from electronic medical records documented in our hospital database and our picture archiving and communication system (PACS).

### 2.3 Statistical analysis

Statistical analyses were performed using SAS version 9.4 (SAS Institute Inc., North Carolina, USA). Group comparisons with respect to bone pain were conducted using the chi-square test for categorical variables and the Wilcoxon two-sample test for continuous variables. These *p*-values are regarded as a descriptive characterization. Continuous variables were analyzed as continuous measures for descriptive purposes and categorized for regression analyses, where appropriate.

For the primary aim of assessing risk factors for bone pain, a comprehensive set of potential predictors was considered based on clinical relevance and prior literature.

Subsequently, univariate logistic regression models were fitted for each candidate predictor to estimate odds ratios (ORs) with 95% confidence intervals (CIs). This univariate regression was used to identify variables eligible for inclusion in the multivariable model.

A multivariable logistic regression model was then constructed to identify independent predictors of bone pain. Variables were selected for multivariable analysis based on clinical relevance and the results of the univariate analyses. Treatment exposure was included as an a priori covariate to control for potential confounding. An initial multivariable model including the preselected candidate variables was fitted, followed by variable selection to derive the final model. Candidate variables for the multivariable model were preselected based on univariate analyses (*p* < 0.2) and clinical relevance to reduce the risk of multicollinearity. Model stability was additionally assessed using forward selection, which resulted in the same final model. The final multivariable model represents the main analytical result of this study, as it identifies those factors that remained independently associated with bone pain after simultaneous adjustment for other relevant covariates. Variables considered in the initial modeling process but not retained in the final model are indicated accordingly in the results tables to ensure transparency of the model-building strategy. The impact of bone pain on PROMs was assessed using the Wilcoxon two-sample test. Statistical significance was defined as *p*-values less than 0.05. When quoting percentages, the figures were rounded to the nearest whole number.

## 3 Results

### 3.1 Patient characteristics and disease burden stratified by bone pain

Baseline demographic and clinical characteristics of the study population are presented in Table 1. A total of 352 patients with MM were included in the analysis. The cohort had a median age of 68 years at study inclusion representing a real-world patient population. Fifty-two percent reported bone pain at study enrollment. They were older compared to patients without bone pain (median 69 vs. 67 years, *p* = 0.242), although the differences did not reach statistical significance. No sex-specific differences were observed between the two groups (*p* = 0.694).

**Table 1:** Patient and disease characteristics.

	All patients *n* = 352 (100%)	Patients with bone pain n = 184 (52%)	Patients without bone pain *n* = 168 (48%)	*p*-value bone pain vs. no bone pain
Age in years at study inclusion, median (range)	68 (41–95)	69 (45–95)	67 (41–90)	0.242
Age in years at ID, median (range)	65 (36–86)	65 (36–84)	65 (36–86)	0.360
Sex, male/female	231 (66%) / 121 (34%)	119 (65%) / 65 (35%)	112 (67%) / 56 (33%)	0.694
CRAB at ID⁎				
CRAB-C	74 (21%)	31 (17%)	43 (26%)	**0.044**
CRAB-R	123 (35%)	53 (29%)	70 (42%)	**0.012**
CRAB-A	207 (59%)	106 (58%)	101 (60%)	0.633
CRAB-B	296 (84%)	165 (90%)	131 (78%)	**0.003**
ISS at ID				0.929
ISS-I	118 (34%)	60 (33%)	58 (35%)	n/a
ISS-II	111 (32%)	59 (32%)	52 (31%)	n/a
ISS-III	123 (35%)	65 (35%)	58 (35.5%)	n/a
Duration of disease in years, median (range)	3 (0–26)	3 (0–24)	3 (0–26)	0.346
Osteolytic lesions with risk of instability	55 (16%)	42 (25%)	13 (11%)	**0.0001**
Progressive disease	73 (21%)	48 (26%)	25 (15%)	**0.001**

⁎ Multiple options possible, CRAB = hypercalcemia, renal failure, anemia, bone lesions. ID = initial diagnosis, ISS = International Staging System, n/a = not applicable.

Patients with bone pain at study inclusion demonstrated a distinct bone-centric disease pattern, characterized by highly significant increases in osteolytic lesions with structural instability risk (25% vs. 11%, *p* = 0.0001) and higher prevalence of CRAB-B already present at ID (90% vs. 78%, *p* = 0.003). In addition, this group showed a significantly higher prevalence of progressive disease (PD) (26% vs. 15%, *p* = 0.001). In contrast, patients without bone pain exhibited a greater non–bone-related disease burden at initial diagnosis, with significantly higher frequencies of CRAB-C (26% vs. 17%, *p* = 0.044) and CRAB-R (42% vs. 29%, *p* = 0.012).

Overall disease burden at initial diagnosis was substantial: CRAB-C 21%, CRAB-R 35%, CRAB-A 59%, and CRAB-B 84%.

### 3.2 Pre-existing orthopedic conditions

Pre-existing orthopedic conditions, based on patient-reported medical history, were documented in 169 of 352 patients (48%) and were significantly more frequent in patients with bone pain at study inclusion (60% vs. 35%; *p* < 0.0001).

Patients with bone pain more frequently reported prior orthopedic diagnoses of nonspecific low back pain (23% vs. 11%; *p* = 0.027), sciatica (9% vs. 2%; *p* = 0.007), and spinal stenosis (11% vs. 4%; *p* = 0.018). Notably, these pre-existing diagnoses did not consistently correspond with current bone pain, as 14% of patients without bone pain at study inclusion had a history of low back pain, 2% of sciatica, and 4% of spinal stenosis. Cervical disc herniation was more prevalent in patients with bone pain (8% vs. 2%; *p* = 0.011), while overall disc herniation and thoracic/lumbar disc herniation showed no significant differences.

Degenerative joint disease was markedly enriched in the bone pain group, including gonarthrosis (27% vs. 3%; *p* < 0.0001), coxarthrosis (19% vs. 6%; *p* = 0.0002), and omarthrosis (11% vs. 3%; *p* = 0.003). Pre-existing vertebral fractures were reported by 34% of all patients and occurred significantly more frequently in the bone pain group (14% vs. 5%; p = 0.003), predominantly affecting the lumbar spine (11% vs. 4%; *p* = 0.012). Pre-existing orthopedic conditions are shown in Table 2.

**Table 2:** Preexisting orthopedic condition(s).

Pre-existing orthopedic condition⁎	All patients n = 352 (100%)	Patients with bone pain n = 184 (52%)	Patients without bone pain n = 168 (48%)	p-value
Total (%)	169 (48%)	111 (60%)	58 (35%)	**<0.0001**
Lumbar back pain	65 (19%)	42 (23%)	23 (14%)	0.027
Sciatica	21 (6%)	17 (9%)	4 (2%)	**0.007**
Gonarthrosis	54 (15%)	29 (27%)	5 (3%)	**<0.0001**
Coxarthrosis	45 (13%)	35 (19%)	10 (6%)	**0.0002**
Spinal stenosis⁎⁎	27 (8%)	20 (11%)	7 (4%)	**0.018**
thoracic	5 (1%)	3 (2%)	2 (1%)	0.728
lumbar	23 (7%)	17 (9%)	6 (4%)	**0.032**
Disc herniation⁎⁎	48 (14%)	31 (17%)	17 (10%)	0.066
cervical	17 (5%)	14 (8%)	3 (2%)	**0.011**
thoracic	4 (1%)	3 (2%)	1(1%)	0.360
lumbar	39 (11%)	25 (14%)	14 (8%)	0.117
Osteoporosis	27 (8%)	18 (10%)	9 (5%)	0.119
Vertebral fracture⁎⁎	34 (10%)	26 (14%)	8 (5%)	**0.003**
cervical	4 (1%)	3 (2%)	1 (1%)	0.360
thoracic	10 (3%)	7 (4%)	3 (2%)	0.255
lumbar	28 (8%)	21 (11%)	7 (4%)	**0.012**
Omarthrosis	26 (7%)	21 (11%)	5 (3%)	**0.003**
Rheumatoid arthritis	2 (1%)	2 (1%)	0 (0%)	0.175

⁎ Multiple answers possible.

⁎⁎ Multiple spinal regions may be involved, therefore sum >100%.

### 3.3 Localization and cause of bone pain

**Fig. 1:**
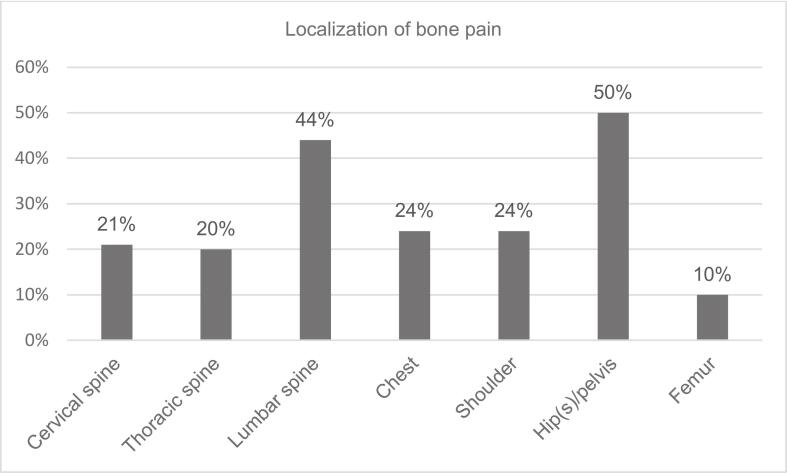
Localization of bone pain in all patients with bone pain (*n* = 184). Multiple answers possible, therefore in sum >100%.

The anatomical distribution of bone pain showed predominant involvement of the hip(s)/pelvis (50%) and lumbar spine (44%). Further details are depicted in Fig. 1.

Among the 184 patients reporting bone pain at study inclusion, correlation analysis between self-reported pain localization and current computed tomography findings was performed. In 130 patients (71%), the anatomical distribution of bone pain was consistent with radiologically documented osteolytic lesions at the corresponding sites, indicating myeloma-specific skeletal involvement. In the remaining 54 patients (29%), the reported bone pain localization did not correspond with osteolytic lesions on current imaging.

### 3.4 Patient-reported outcome measures (PROMs)

The median QoL and health-related status for patients with bone pain was significantly lower compared to patients without bone pain (4 vs. 5; *p* < 0.001), representing a difference from neutral to fairly good (Table 3).

**Table 3:** The impact of bone pain on patient-reported outcome measures.

PROMs	All patients n = 352 (100%)	Patients with bone pain n = 184 (52%)	Patients without bone pain n = 168 (48%)	p-value
Quality of life	5 (1–7)	4 (1–7)	5 (1–7	<0.001*
Health-related status	5 (1–7)	4 (1–7)	5 (1–7)	**<0.001**

PROMs = patient-reported outcome measures. 1 = very poor, 2 = poor, 3 = fairly poor, 4 = neutral, 5 = fairly good, 6 = good, 7 = excellent. Data are presented as median (range).

### 3.5 Predictors of bone pain

Potential risk factors for bone pain were evaluated in univariate analysis. Factors associated with a significantly increased risk of bone pain were pre-existing orthopedic conditions, CRAB-B at ID, osteolytic lesions at acute risk of pathological fracture, and PD. The initial multivariate logistic regression model included the following variables: current therapy, age at study inclusion and at ID, sex, pre-existing orthopedic conditions, CRAB, ISS stage, disease duration, osteolytic lesions at acute risk of pathological fracture, and PD. Using multivariate backward selection, pre-existing orthopedic conditions remained independently associated with bone pain (OR 2.78, 95% CI 1.77–4.36; *p* < 0.0001), as did the presence of osteolytic lesions at acute risk of pathological fracture (OR 3.06, 95% CI 1.46–6.40, *p* = 0.003). Patients with CRAB-B at ID (OR 1.74, 95% CI 0.92–3.29, *p* = 0.09) and PD (OR 1.68, 95% CI 0.93–3.05, p = 0.09) showed an increased, although not statistically significant risk of bone pain. No significant associations were observed for current therapy, age, sex, CRAB-C/-R/-A, ISS stage, and disease duration (Table 4).

**Table 4:** Univariate and multivariate logistic regression analysis with backward selection predicting the risk of bone pain in patients with Multiple Myeloma.

	Univariate Logistic regression			Multivariate Logistic regression with backward selection		
	Odds ratio	95% CI	p-value	Odds ratio	95% CI	p-value
*Patient's characteristics*						
Age (>70 years)	1.47	0.96–2.24	0.073	n/a	n/a	n/a
Age at ID (>65 years)	1.27	0.83–1.93	0.266	n/a	n/a	n/a
Sex female vs. male	1.09	0.70–1.70	0.694	n/a	n/a	n/a
Orthopedic conditions	2.88	1.87–4.45	<0.0001	2.77	1.77–4.36	**<0.0001**

*Current therapy*						
IMiDs	0.99	0.65–1.52	0.973	n/a	n/a	n/a
Immunotherapy	1.44	0.93–2.22	0.100	n/a	n/a	n/a
PI + BP or Den	1.31	0.85–2.02	0.229	n/a	n/a	n/a

*Disease characteristic*						
CRAB at ID*						
CRAB-C	0.59	0.35–0.99	0.046	n/a	n/a	n/a
CRAB-R	0.57	0.36–0.88	0.012	n/a	n/a	n/a
CRAB-A	0.90	0.59–1.38	0.633	n/a	n/a	n/a
CRAB-B	2.45	1.35–4.46	0.003	1.74	0.92–3.29	0.09

ISS at ID						
ISS 3 vs. 1	1.08	0.65–1.80	0.880	n/a	n/a	n/a
ISS 2 vs. 1	1.10	0.65–1.80	0.820	n/a	n/a	n/a
Duration of disease ≥3 years	1.16	0.76–1.76	0.499	n/a	n/a	n/a
Osteolytic lesions at risk of pathological fracture**	3.50	1.81–6.80	0.0002	3.06	1.46–6.40	**0.003**
Progressive disease	2.02	1.18–3.46	<0.0001	1.68	0.93–3.05	0.09

All variables considered as predictors are shown in the univariate logistic regression (left); Variables retained after backward selection are presented in the multivariate logistic regression model (right). *multiple criteria possible ** in imaging not older than three month. IMiDs = Immunomodulatory Imide Drugs, PI = Proteasome Inhibitors, BP = Bisphosphonates, Den = Denosumab CRAB = hypercalcemia, renal failure, anemia, bone lesions. ID = initial diagnosis, ISS = International Staging System. n/a = not applicable.

## 4 Discussion

This prospective study of 352 MM patients identified osteolytic lesions with fracture risk (OR 3.06, *p* = 0.003) and pre-existing orthopedic conditions (OR 2.77, *p* < 0.0001) as independent predictors of bone pain. Bone pain affected more than half of the cohort and significantly reduced QoL. Traditional prognostic factors including ISS stage and CRAB criteria were not independent predictors, indicating that bone pain is driven by both myeloma-related skeletal disease and coexisting orthopedic conditions, requiring careful differentiation for appropriate therapy.

### 4.1 Clinical and disease characteristics

The cohort, with median age of 68 years and disease duration of 3 years, had comparable baseline characteristics between groups. Bone disease (CRAB-B) at ID was present in 84%, which is consistent with literature [7], [14]. While CRAB-B at ID showed a twofold increased risk of bone pain, this association did not remain significant after adjustment.

The relatively high prevalence of CRAB-C in our overall cohort (21%) compared with the approximately 10% reported in population-based cohorts [15] likely reflects the referral character of a tertiary university center. This pattern was confirmed in a comprehensive analysis of younger patients, which reported a 19% prevalence of CRAB-C in tertiary center setting. The higher CRAB prevalence suggested a more aggressive disease phenotype and symptom burden at ID. Importantly, this finding was not associated with a worse prognosis per se [16]. Neither sex (*p* = 0.96) nor age (*p* = 0.07) predicted bone pain in multivariate analysis, despite literature suggesting younger age and female sex influence pain prevalence [17], [18], [19], [20]. The ISS stage is used to distinguish prognostic subgroups and is based on two parameters: β2-microglobulin and serum albumin. Elevated β2-microglobulin levels are associated with increased tumor burden, while reduced albumin levels reflect inflammatory cytokine activity within the myeloma microenvironment [21]. ISS stage distribution was comparable between groups and did not predict bone pain, suggesting that MM-related pain is not driven solely by disease severity, possibly due to symptom control by ongoing therapy.

### 4.2 Anatomical distribution and pain etiology

Bone pain in MM patients showed a heterogeneous anatomical distribution with a clear predominance of axial and proximal skeletal sites, reflecting typical marrow-rich bone involvement in multiple myeloma [22]. Radiological correlation revealed that in 71% of patients with bone pain (130/184), the reported pain localization corresponded to morphologically detectable osteolytic lesions on CT imaging, consistent with myeloma-related skeletal involvement. In the remaining 29% (54/184), no corresponding CT-detectable osteolytic lesion was identified at the reported pain site. This discordance may reflect concomitant non-malignant musculoskeletal or orthopedic conditions but may also be explained by myeloma manifestations that are not yet morphologically apparent on CT, such as early bone marrow infiltration or microfractures. In our cohort pre-existing orthopedic conditions were significantly more prevalent in patients with bone pain (60% vs. 35%; *p* < 0.0001) and emerged as an independent predictor of bone pain (OR 2.77, p < 0.0001). Conditions included lumbago (23%) and sciatica (9%), while bone pain most commonly affected the lumbar spine (44%) and hips/pelvis (50%). The spine is consistently reported as the most frequently affected site of myeloma-related vertebral compression fractures [23]. Although the etiology of the vertebral fractures (10%) is not documented in detail, it can be assumed that they were pathological fractures related to myeloma-bone disease and/or concomitant osteoporosis.

The observed association between pre-existing orthopedic conditions and bone pain is of particular clinical interest. Pre-existing musculoskeletal disease may amplify MM pain trough neuroplasticity mechanism [24], however the cross-sectional design of the present study does not permit conclusions regarding the underlying cause of this association. It remains unclear whether patients with pre-existing orthopedic disease have an increased susceptibility to musculoskeletal symptoms, whether these conditions contribute to persistent pain after myeloma-related skeletal events, or whether overlapping clinical manifestations account for the observed relationship. Nevertheless, our findings identify patients with pre-existing orthopedic conditions as a clinically vulnerable subgroup with an increased likelihood of experiencing bone pain. From a clinical perspective, this supports careful interdisciplinary evaluation to distinguish myeloma-related from non-myeloma-related pain and may facilitate risk stratification and individualized supportive care. The findings underscore that bone pain in multiple myeloma is frequently multifactorial and should not automatically be assumed to reflect active myeloma bone disease.

### 4.3 Impact of bone pain on patient-reported outcome measures

Patients with bone pain demonstrated significantly lower QoL and worse health-related status. Consistent with literature, we identified pain as a key driver to lower QoL [11], [25], [26].

### 4.4 Independent predictors of bone pain

In the multivariate logistic regression model, four independent variables were associated with an increased risk of bone pain, and two remained statistically significant after backward selection. Occurrence of osteolytic lesions at acute risk of pathological fractures emerged as the strongest predictor, tripling the odds of bone pain (OR 3.06, *p* = 0.003). Bone destruction can cause pain through multiple mechanisms: periosteal nerve sprouting, microfractures, mechanical instability, and inflammatory mediator release [27], [28], [29]. This finding supports current recommendations for bone-directed therapy in patients with myeloma bone disease [29], [30], [31].

Pre-existing orthopedic conditions were also found to be an independent predictor of bone pain (OR 2.78, *p* < 0.0001), emphasizing the challenge of differentiating pain etiologies.

Patients with CRAB-B at ID (OR 1.74) showed a possible, yet not statistically significant, increased risk of bone pain. This observation is likely due to the fact that myeloma bone lesions rarely heal due to decreased osteoblast activity [32], [33]. Consequently, in patients who already have bone lesions at ID, bone pain may persist as a chronic condition throughout the course of the disease. This concept is supported by our previous data [7] demonstrating bone pain in 74% of patients at ID, 47% of patients with stable disease, and 66% of patients with PD. The significantly higher prevalence in PD patients (OR 1.9, *p* = 0.0126) suggested that PD may exacerbate pain symptoms. Furthermore, in our backward selection model, PD (OR 1.68) showed a trend toward independently predicting bone pain, though this association did not reach statistical significance. Together, these findings suggest a complex, potentially bidirectional relationship between bone pain and PD in MM. No association were observed between bone pain and ISS stage or disease duration, suggesting that MM-related bone pain is not driven solely by advanced disease severity.

### 4.5 Strengths and limitations

Strengths of this study include the prospective design, large sample size, comprehensive assessment of multiple potential predictors, and inclusion of PROMs. The interdisciplinary approach reflects real-world clinical practice and the median age of 69 years represents real-life population. The study carries limitations due to its single tertiary-center design, which limits generalizability. The assessment of bone pain at a single time point limits the ability to analyze pain trajectories and temporal relationships with potential predictors. As questionnaires were administered irrespective of treatment phase, disease status, imaging time points, or skeletal events, the study provides a cross-sectional snapshot rather than a longitudinal evaluation of bone pain throughout the disease course. Consequently, the observed associations should not be interpreted as causal and may be influenced by the dynamic nature of multiple myeloma and its treatment. Moreover, the influence of specific myeloma therapies on bone pain was not a primary objective of the present study, which focused on identifying clinical factors and pre-existing musculoskeletal comorbidities associated with bone pain. The effects of individual therapies should therefore be addressed in future longitudinal studies incorporating repeated pain assessments at standardized clinical time points. The PROM instrument used adapted two global items from the EORTC QLQ-C30 (overall health status and quality of life) but did not employ the complete validated questionnaire, limiting strict comparability with other studies. A further limitation is that the variable “osteolytic lesions at risk of instability” was derived from routine clinical radiological assessment rather than from a dedicated standardized imaging review. Consequently, no formal instability score or inter-reader agreement analysis was available. Although imaging was assessed within an experienced institutional myeloma imaging workflow, residual operator dependence and variability cannot be excluded. Additionally, the etiology of vertebral fractures (osteoporotic, myeloma-related, or traumatic) was not systematically assessed.

### 4.6 Future directions

Prospective cohort studies with serial pain evaluations are needed to clarify distinct pain trajectories, ascertain temporal associations between predictors and pain progression, and assess the influence of specific interventions, particularly bone-modifying agents.

## 5 Conclusion

This large prospective study demonstrates that bone pain in multiple myeloma is a multifactorial condition affecting more than half of patients and significantly impairing quality of life. Osteolytic lesions with fracture risk and pre-existing orthopedic conditions were independently associated with the presence of bone pain, while traditional disease severity markers were not independently associated. Notably, nearly one-third of bone pain did not correlate with myeloma-related skeletal lesions, highlighting that bone pain cannot always be attributed to CT-visible osteolytic lesions alone. These findings emphasize the challenge of distinguishing between myeloma-related pain and bone pain associated with unspecific causes or pre-existing orthopedic conditions. They further underscore the importance of interdisciplinary collaboration between oncology, radiology and orthopedics to ensure accurate diagnosis and individualized, targeted treatment.

Disclosures

CP, GWH, GI, JPM, INS and HS have nothing to disclose. RW reports honoraria from Pfizer, Amgen, Novartis, Sanofi, Janssen, BMS, and Kite/Gilead, outside the submitted work. ME reports grants and personal fees from Amgen, Janssen, BMS, Takeda, outside the submitted work. ET reports honoraria from BMS, Takeda, Menarini/Stemline, Janssen, GSK, EUSA Pharma, ASTRA/Zeneca, Amgen, Pfizer, Sanofi and travel expenses from Takeda, EUSA Pharma, Astra/Zeneca, Amgen, and Sanofi outside the submitted work.

## Use of artificial intelligence

The authors used ChatGPT (OpenAI) to assist with language editing, including grammar, spelling, and improving the readability of the manuscript. No scientific content, data analysis, or interpretation was generated using artificial intelligence.

## CRediT authorship contribution statement

**Carlotta Pietsch:** Writing – review & editing, Writing – original draft, Methodology, Investigation, Formal analysis, Conceptualization. **Monika Engelhardt:** Writing – review & editing, Writing – original draft, Methodology, Investigation, Formal analysis, Conceptualization. **Julian P. Maier:** Writing – review & editing, Writing – original draft, Methodology, Conceptualization. **Gabriele Ihorst:** Writing – review & editing, Formal analysis. **Hagen Schmal:** Writing – review & editing. **Evangelos Terpos:** Writing – review & editing. **Ioannis Ntanasis-Stathopoulos:** Writing – review & editing. **Ralph Wäsch:** Writing – review & editing, Investigation, Formal analysis. **Georg W. Herget:** Writing – review & editing, Writing – original draft, Methodology, Investigation, Formal analysis, Conceptualization.

## Ethics approval and consent to participate

Approval for this study was obtained from the local ethics committee of the Albert-Ludwigs-University Freiburg (EK 30/18_250091).

## Declaration of competing interest

The authors declare that they have no known competing financial interests or personal relationships that could have appeared to influence the work reported in this paper.
